# Cushing’s Syndrome With Acute Psychosis: A Case Report

**DOI:** 10.7759/cureus.25761

**Published:** 2022-06-08

**Authors:** Abdullelah S Alfakhri, Meshal Alaqeel, Mansour I Alnasir

**Affiliations:** 1 Psychiatry, King Fahad Medical City, Riyadh, SAU; 2 Medicine, King Saud Bin Abdulaziz University for Health Sciences, Riyadh, SAU; 3 Psychiatry, Eradh Comlex, Riyadh, SAU

**Keywords:** cushing’s disease with acute psychosis, cushing’s syndrome with acute psychosis, psychiatry case report, acute psychosis, cushing’s disease, cushing’s syndrome

## Abstract

Cushing syndrome is a rare disease that rarely presents as acute psychosis. In this case, the patient presented with acute psychosis and agitation as the first manifestations of the disease which led to the admission of the patient to a psychiatry hospital for one month, as it was difficult to restrain her sufficiently for performing appropriate diagnostic tests due to disturbing behavior. She responded well to treatment with olanzapine and lorazepam to treat the patient’s agitation, and successfully complete her evaluation. Thereafter, she was diagnosed with a pituitary tumor and underwent pituitary lesion resection via a microscopic transsphenoidal as needed. Two months after surgery, her cortisol levels returned to baseline, and she became calmer and decreased the tensity of her psychosis; however, it was only five months after surgery that her psychotic symptoms and disturbed behavior ceased.

## Introduction

Cushing syndrome is comprised of a group of symptoms induced by prolonged exposure to high blood cortisol levels [1]. It is a rare disease, occurring in approximately 2.4 per million individuals per year [2]. Psychiatric and cognitive manifestations of Cushing syndrome occur in 70%-85% of patients, with irritability, emotional lability, and depression occurring most commonly. Rarer symptoms include mania, panic attacks, anxiety, suicidal ideation, and acute psychosis [3-5]. In this article, we describe a patient with Cushing syndrome who developed psychosis with agitation as the first manifestation of Cushing syndrome. The patient was difficult to manage since her agitation and refusal to undergo evaluation prevented her from receiving outpatient care.

## Case presentation

A 22-year-old woman with a three-month history of an increase in appetite, binge eating, and weight gain. After two weeks of her initial symptoms, she started to have grandiose and persecutory delusions, auditory hallucinations, decreased need for sleep, agitation, irritability, and aggression for which she went to a private psychiatry clinic and was given 10 mg olanzapine oral at night. After a month of starting oral olanzapine, she was not improving and was admitted to the psychiatry ward for evaluation. During her admission period, she started to have cognitive symptoms including worsened memory, attention, and orientation. After one month of admission with no improvement on medication, she was noted to have moon face and high blood pressure, and her laboratory investigation showed mild hypokalemia, high cortisol level, and adrenocorticotropic hormone (ACTH), elevated liver enzymes, and mild hypertriglyceridemia. A magnetic resonance imaging (MRI) scan of the brain revealed a 6 × 2-mm hyperintense lesion in the anterior pituitary on a T2-weighted image; therefore, she was transferred to our hospital for further work up and management as we have the endocrine facility. She had no past psychiatric history or family history of psychiatric illnesses, nor a history of substance abuse. She also had no past medical history and was not on any medication prior to this presentation.

The patient was admitted to the endocrine department to evaluate the possibility of Cushing syndrome. Her blood pressure (150/98), heart rate (128 BPM), and respiratory rate (30 BPM) were elevated. She was treated with losartan, amlodipine, and spironolactone. Basic labs were done (Table 1). Therefore, insulin therapy was initiated. The evaluation of the patient’s condition was difficult as she was aggressive and uncooperative due to a lack of insight. Her primary team planned for sedation with anesthesia to facilitate a clinical evaluation; however, no intensive care unit bed was available.

**Table 1:** Lab results for the patient when she first came to our hospital

Lab test	Patient result	Reference values
cortisol levels	1549 nmol/L	140 to 690 nmol/L
ACTH (Adrenocorticotropic Hormone)	54 pg/mL	10 to 50 pg/mL
ALT (Alanine transaminase)	305 U/L	7 to 56 U/L
AST (Aspartate aminotransferase)	112 U/L	8 to 33 U/L
Alkaline phosphatase	141 IU/L	44 to 147 IU/L
Hemoglobin A1c	7.3%	5.7% to 6.4%

**Figure 1:**
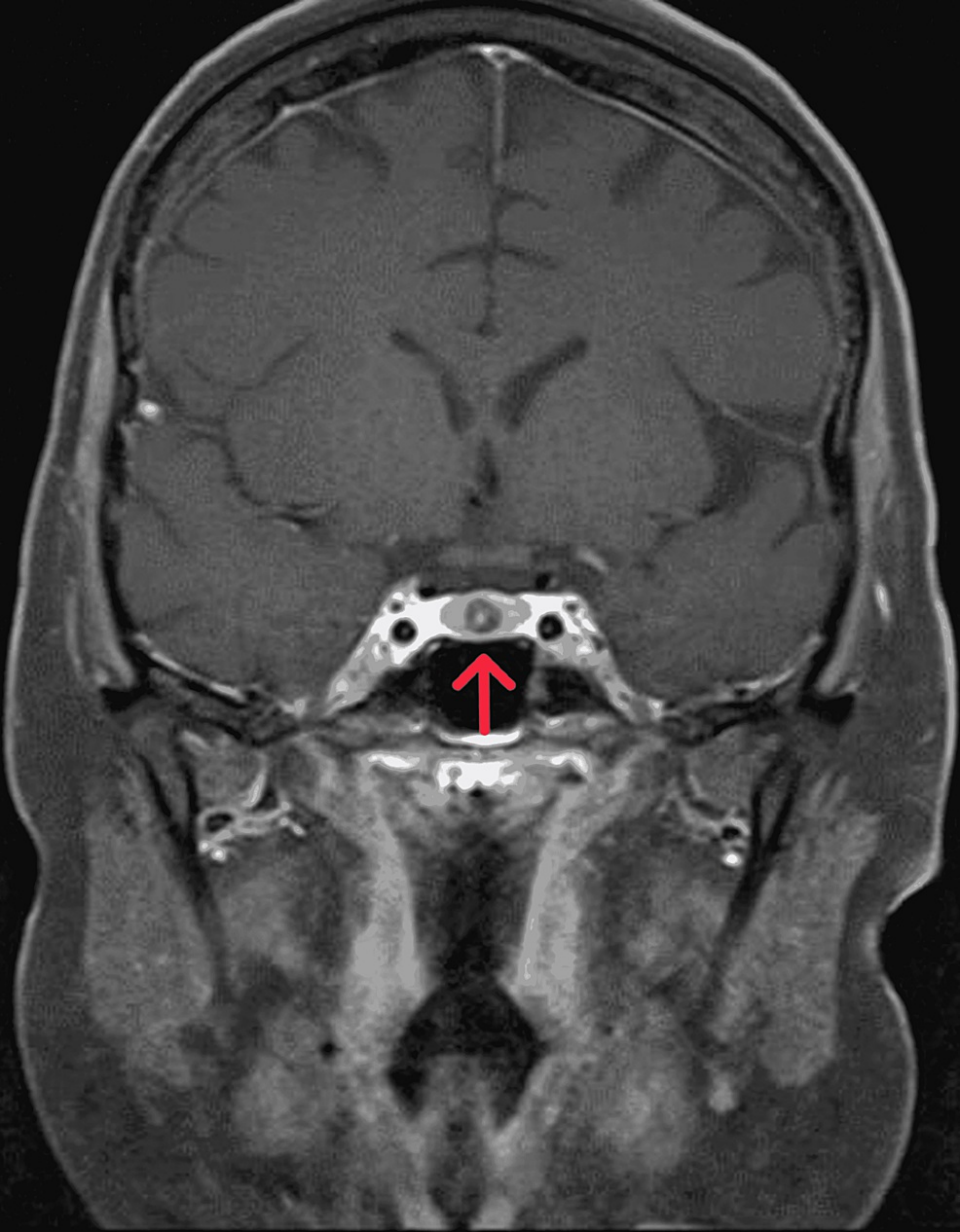
Coronal T1-weighted MRI of the pituitary gland with contrast showed a hypoenhancing nodular lesion at the midline of the anterior pituitary, with mild eccentric to the right

**Figure 2:**
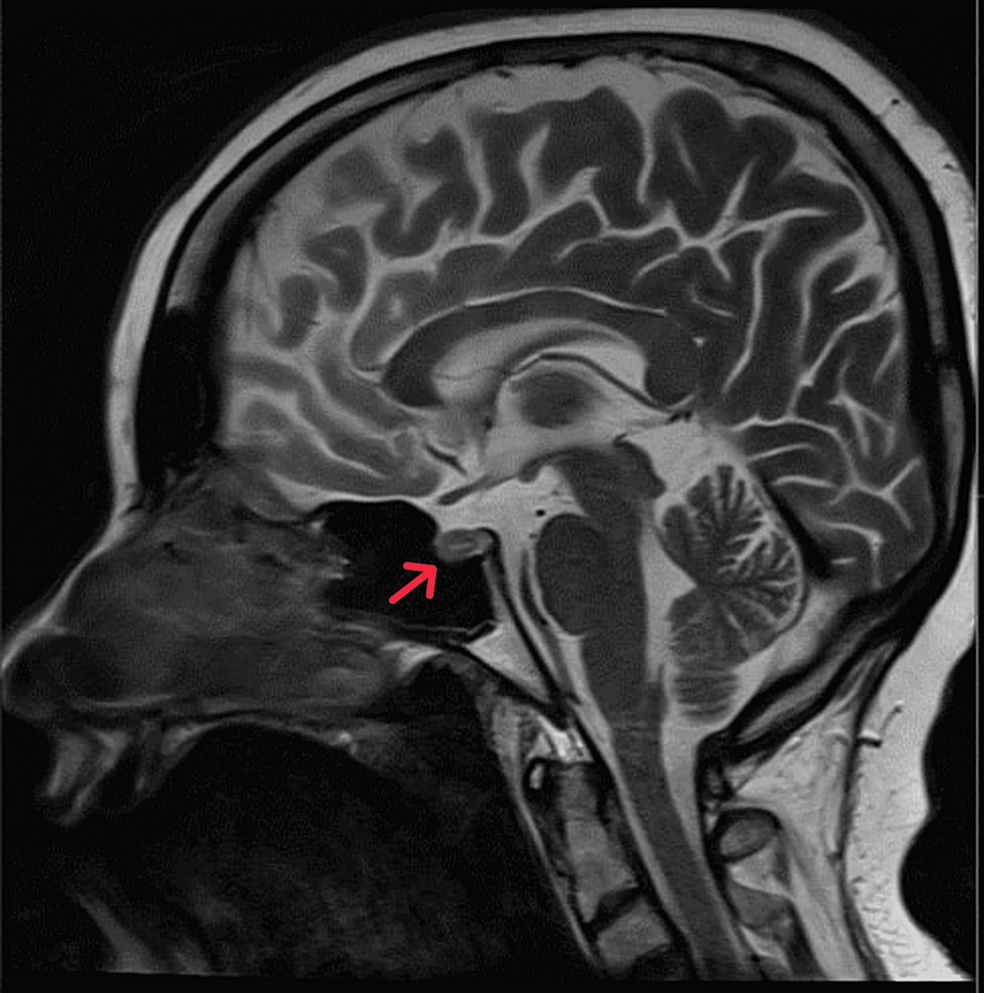
Brain MRI sagittal view showing focal anterior pituitary hypoenhancing lesion at the midline and eccentric to the right

Psychiatry was consulted to manage agitation. We started her on 5 mg olanzapine oral twice daily, and 2 mg lorazepam three times daily intravenous when oral was not possible. Maximum dosage of 5 mg olanzapine and 2 mg lorazepam every four hours were administered as required to manage agitation. Her ECG showed a QTC of 464. One-to-one nurse observation was initiated to detect risky behaviors. The patient slept well and became calmer and more cooperative throughout evaluations when receiving medication. One-to-one nurse observation was discontinued after five days, and lorazepam administration was reduced to two times daily. She remained easily provoked with grandiose and persecutory delusions, auditory hallucinations, and confusion. As the patient calmed, the primary team continued clinical evaluations. A contrast-enhanced MRI showed a focal non-deforming and hypo-enhancing lesion, measuring 7 mm (AP) x 6 mm (TV) x 6 mm (CC), in the anterior pituitary (Figures 1, 2). A minimal leftward deviated pituitary stalk with normal thickness was also identified. An 8 mg dexamethasone suppression test revealed cortisol levels had decreased from 1,500 to 900 nmol/L. The 24-hour cortisol level was not determined, as the patient was easily provoked. Inferior petrosal sinus sampling was performed under general anesthesia. These results are consistent with central Cushing disease.

Treatment with 250 mg metyrapone twice daily was initiated and the patient was scheduled for pituitary lesion resection via a microscopic transsphenoidal approach by neurosurgery. Her blood tests began normalizing post-surgery except for low cortisol (Table 2), and her vital signs were within normal range. Medications regulating blood pressure and glucose levels were decreased to monotherapy and discontinued thereafter. And 40 and 20 mg doses of hydrocortisone administered in the morning and night, respectively, were tapered to 5 mg twice daily over a period of two months after the surgery, and cortisol levels were regulated reaching 167 nmol/L. Agitation and irritability, grandiose and persecutory delusion and auditory hallucination tensity were reduced, with intact cognitive and memory function. Therefore, medication dosages were gradually reduced, starting with lorazepam.

**Table 2:** Lab results after the surgery.

Lab Test	Patient result	Reference values
cortisol levels	68 nmol/L	140 to 690 nmol/L
ACTH (Adrenocorticotropic Hormone)	25 pg/ml	10 to 50 pg/mL
ALT (Alanine transaminase)	17.2 U/L	7 to 56 U/L
AST (Aspartate aminotransferase)	19.2 U/L	8 to 33 U/L
Alkaline phosphatase	121 IU/L	44 to 147 IU/L
TSH (Thyroid Stimulating Hormone)	1.8 mIU/L	0.5 to 5.0 mIU/L

Before discharge, the patient’s psychotropic medications were withheld by the primary team for two days due to oversedation. Upon discharge, due to the side effects of olanzapine, the patient was switched to oral risperidone 1 mg at night, with 0.5 mg oral clonazepam twice daily as needed for agitation and psychosis. Throughout follow-up, the patient experienced ongoing psychosis with disturbed behavior even though she is using received clonazepam twice daily. Therefore, her dosage of risperidone was increased to 2 mg orally at night, and oral clonazepam (0.5 to 1 mg) was administered three times daily as needed to manage agitation. After three months of discharge (five months from surgical intervention), her levels of agitation and irritability decreased, delusions and auditory hallucinations ceased, and she returned to baseline, and clonazepam was discontinued and risperidone dosage was tapered to 0.5 mg with observation and follow up in the clinic, and no symptom relapse was observed. The complete discontinuation of her medications is planned next visit while monitoring the patient for signs of relapse. 

## Discussion

Cushing syndrome may initially present as psychosis, which may be misdiagnosis as a primary psychotic disorder, delaying the proper diagnosis and management. Our patient presented to a psychiatry hospital before being referred to us because she resisted psychosis treatment, the resistance to treatment of primary illness due to psychiatric manifestation is not uncommon, as Fujii et al. [6] reported the management of a patient who resisted schizophrenia treatment for 10 years before being diagnosed with Cushing syndrome.

Agitation with psychosis is likely the main obstacle for properly evaluating, diagnosing, and treating patients with Cushing syndrome. In our patient, we aimed to reduce her agitation to facilitate clinical evaluation. The organic cause of psychosis often responds poorly to antipsychotic medication and exhibits a challenge in managing agitation which necessitate the utilization of highly sedating medications, to facilitate further clinical evaluation. Shah et al. [7] reported similar difficulty treating a patient with agitation despite prescribing lorazepam and 1 mg haloperidol twice daily, agitation was poorly controlled. In our case, the patient responds to a high dose of Olanzapine with lorazepam in a better way than the case report that was managed with haloperidol with lorazepam.

Psychiatric symptoms secondary to medical conditions usually occur transiently and they resolve after treatment of the primary cause, however, the duration for complete resolution of symptoms is unknown. In our case, the patient gradually improved for three months prior to achieving remission, whereas a patient reported by Wu et al. [8] went into complete remission one-month post-cortisol level correction.

## Conclusions

Cushing syndrome, like many other endocrine diseases, can present as treatment-resistant psychiatric symptoms, which may be missed and treated as a primary psychiatric illness due to the lack of proper assessment and management. In this study, we tried to correlate the psychiatric symptoms with Cushing syndrome, the challenges we faced, and the response to the treatment. Our case report gives an insight into possible rare secondary causes of psychosis and advice a thorough evaluation of patients.
